# Gene-to-gene interactions regulate endogenous pain modulation in fibromyalgia patients and healthy controls—antagonistic effects between opioid and serotonin-related genes

**DOI:** 10.1097/j.pain.0000000000000896

**Published:** 2017-03-07

**Authors:** Jeanette Tour, Monika Löfgren, Kaisa Mannerkorpi, Björn Gerdle, Anette Larsson, Annie Palstam, Indre Bileviciute-Ljungar, Jan Bjersing, Ingvar Martin, Malin Ernberg, Martin Schalling, Eva Kosek

**Affiliations:** a Department of Clinical Neuroscience, Osher Center, Karolinska Institutet, Stockholm, Sweden; b Department of Neuroradiology, Karolinska University Hospital, Stockholm, Sweden; c Department of Clinical Sciences, Karolinska Institutet, Stockholm, Sweden; d Department of Rehabilitation Medicine, Danderyd Hospital, Stockholm, Sweden; e Department of Health and Rehabilitation, Institute of Neuroscience and Physiology, Sahlgrenska Academy, University of Gothenburg, Gothenburg, Sweden; f Pain and Rehabilitation Centre, and Department of Medical and Health Sciences, Linköping University, Linköping, Sweden; g Centre for Person Centered Care (GPCC), University of Gothenburg, Gothenburg, Sweden; h Department of Rheumatology and Inflammation Research, Institute of Medicine, Sahlgrenska Academy, University of Gothenburg, Gothenburg, Sweden; i Department of Dental Medicine, Karolinska Institutet, Scandinavian Center for Orofacial Neurosciences (SCON), Huddinge, Sweden; j Department of Molecular Medicine and Surgery, Karolinska Institutet, Center for Molecular Medicine (CMM), Karolinska University Hospital, Stockholm, Sweden; k Stockholm Spine Center, Lowenstromska Hospital, Upplands Vasby, Sweden.

**Keywords:** Chronic pain, Fibromyalgia, Exercise-induced hypoalgesia, Exercise, Pain inhibition, Functional genetic polymorphisms, 5-HTT, Serotonin transporter, OPRM1, Opioid receptor, 5-HT1a, 5-HT1a receptor

## Abstract

Chronic pain is associated with dysfunctional endogenous pain modulation, involving both central opioid and serotonergic (5-HT) signaling. Fibromyalgia (FM) is a chronic pain syndrome, characterized by widespread musculoskeletal pain and reduced exercise-induced hypoalgesia (EIH). In this study, we assessed the effects of 3 functional genetic polymorphisms on EIH in 130 patients with FM and 132 healthy controls. Subjects were genotyped regarding the mu-opioid receptor (OPRM1) gene (*rs1799971*), the serotonin transporter (5-HTT) gene (*5-HTTLPR/rs25531*), and the serotonin-1a receptor (5-HT1a) gene (*rs6296*). The patients with FM had increased pain sensitivity and reduced EIH compared with healthy controls. None of the polymorphisms had an effect on EIH on their own. We found significant gene-to-gene interactions between OPRM1 x 5-HTT and OPRM1 x 5-HT1a regarding activation of EIH, with no statistically significant difference between groups. Better EIH was found in individuals with genetically inferred strong endogenous opioid signaling (OPRM1 G) in combination with weak 5-HT tone (5-HTT low/5-HT1a G), compared with strong 5-HT tone (5-HTT high/5-HT1a CC). Based on the proposed mechanisms of these genetic variants, the findings indicate antagonistic interactions between opioid and serotonergic mechanisms during EIH. Moreover, despite different baseline pain level, similar results were detected in FM and controls, not supporting an altered interaction between opioid and 5-HT mechanisms as the basis for dysfunction of EIH in patients with FM. In summary, our results suggest that, by genetic association, the mu-opioid receptor interacts with 2 major serotonergic structures involved in 5-HT reuptake and release, to modulate EIH.

## 1. Introduction

Chronic pain is associated with an aberrant cerebral response to painful stimuli and dysfunctional pain modulation in particular, which is well characterized in the chronic pain disease fibromyalgia (FM).^19,24^ A proposed mechanism for the pathophysiology of FM is overactivation of endogenous opioid mechanisms, gradually developing into persisting aberrations of pain modulation.^2,16,18^ This indicates that patients with high efficacy of endogenous opioids would have more pronounced dysfunction of pain inhibitory mechanisms. Patients with FM also have a dysregulation of central serotonergic (5-HT) metabolism, with reports of reduced 5-HT metabolites in the cerebrospinal fluid.^36^ Moreover, there is evidence of an antagonistic relationship between opioid and serotonergic mechanisms in regard to pain regulation. The bimodal pain response of opioid agonists with initial analgesia followed by a delayed onset of hyperalgesic effects^8,13^ has also been attributed to 5-HT1a agonists but with a converse effect—initial hyperalgesia followed by a delayed long-term analgesic effect. Interestingly, 5-HT1a agonists have been shown to prevent and reverse opioid-induced hyperalgesia.^3,9^

Genetic association studies offer an intriguing way to study pain-related behavior in vivo in a noninvasive and noninterfering manner in humans. As FM has demonstrated a clear genetic component,^1^ we set out to investigate the interactions between functional polymorphisms of genes coding for key structures involved in opioid and serotonergic signalling. First, the single-nucleotide polymorphism (SNP) *rs1799971* in the OPRM1 gene, regulating the activation of the mu-opioid receptor,^15^ where the presence of the G-allele is proposed to increase the tone of the endogenous opioid system compared with the homozygous AA-genotype.^31,33^ Second, 2 functional polymorphisms (*5-HTTLPR* and *rs25531*) in the serotonin transporter (5-HTT) gene SLC6A4 have jointly been shown to alter the degree of gene expression into high, intermediate, and low expression of 5-HTT.^27,44^ Third, the SNP *rs6295* in the *HTR1A* gene, regulating the expression of the 5-HT1a receptor, where the G-allele, has been linked to reduce the overall 5-HT tone compared with the homozygous CC-genotype.^37,46^ The 5-HTT low-expression genotype has been associated with downregulation of 5-HT1a receptors,^12,30^ indicating that the 5-HTT low expression and 5-HT1a-G genotypes both mediate pain modulation comparably.^29^

The aim of this study was to investigate the interactions between genetically inferred opioid and serotonergic mechanisms on a pain assessment in patients with FM and in healthy controls (HC). We chose exercise-induced hypoalgesia (EIH) as pain induction because it often exacerbates pain and has previously been reported to be dysfunctional in patients with FM.^24,34^ We hypothesized that individuals with genetically inferred strong endogenous opioid mechanisms would have better EIH if they also had weak 5-HT mechanisms (OPRM1 G and 5-HTT low/5-HT1a G) and vice versa (OPRM1 AA and 5-HTT-high/5-HT1a CC). Given our hypothesis that patients with FM have overactivated endogenous opioid mechanisms in combination with 5-HT dysfunction, we hypothesized that FM patients with genetically inferred weaker opioid mechanisms in combination with stronger 5-HT mechanisms (OPRM1-AA and 5-HTT-high/5-HT1a CC) would have more pronounced EIH function.

## 2. Materials and methods

### 2.1. Participants

Female patients with FM and HC of 20 to 65 years were recruited, by newspaper advertisement in the local newspapers of 3 cities in Sweden (Gothenburg, Stockholm, and Linköping), to participate in a 15-week resistance exercise intervention trial (ClinicalTrials.gov identification number: NCT01226784).^25^ Baseline data from this trial are used in this study. Only women were recruited for the study to represent the patient population.^39^ A total of 415 women with FM were screened by telephone for possible eligibility and information about the study. Out of these, 177 women were referred to a medical examination by experienced physicians who performed a standardized interview and palpation of tender points to verify the American College of Rheumatology 1990 criteria for FM.^45^ A total of 52 women were found not eligible because of not meeting the inclusion criteria (n = 32) or declining participation (n = 15). Exclusion criteria were high blood pressure (>160/90 mm Hg), osteoarthritis in hip or knee, other severe somatic or psychiatric disorders, other primary causes of pain than FM, high consumption of alcohol (Audit >6), participation in a rehabilitation program within the past year, regular resistance exercise or relaxation exercise twice a week or more, inability to understand or speak Swedish, and not being able to refrain from analgesics, nonsteroidal anti-inflammatory drugs, or hypnotics for 48 hours before examinations. For HC, 189 women were recruited by telephone screening to complete the baseline assessment, of which 55 women were excluded because of not meeting the inclusion criteria or declining participation. A total of 130 patients with FM and 134 HC, all white women, were eligible and included in the study. Because of inaccurate genotyping (FM n = 4, HC n = 4) and incomplete assessment of EIH (FM n = 4, HC n = 1), the analysed cohort consisted of 122 patients with FM and 129 HC. The study was conducted in accordance with the Declaration of Helsinki, with approval from the regional ethics committee in Stockholm (2010/1121-31/3). All participants were given written and oral information and written consent was obtained.

### 2.2. Procedures

The study took place at 3 sites (Stockholm, Gothenburg, and Linköping), and 4 different investigators performed the test procedures. The investigators had been jointly trained to ensure consistent procedures across the sites. All participants completed standardized questionnaires regarding health status, namely, Hospital Anxiety and Depression Scale (HADS),^6^ Short-Form—36 Bodily Pain Scale (SF-36 BP),^10^ and subjects with FM also completed the Fibromyalgia Impact Questionnaire (FIQ).^5^ HADS is a self-reported scale designed to screen for the presence of anxiety and depression in nonpsychiatric patients. It consists of 2 subscales assessing anxiety (HAD-A) and depression (HAD-D) independently. Each subscale includes 7 assertions with accumulated scores between 0 and 21, where cut-off scores of above 8 can be regarded as the presence of anxiety and depressive disease.^6^ The SF-36 BP is a subscale of the Short-Form 36 (SF-36), a generic health survey for the evaluation of perceived health and functional status. The subscale is a validated instrument to assess pain severity and its interference with working activities over a longer period of time (4 weeks).^17^ The raw scores are transformed into a 0 to 100 scale, where lower scores reflect more pain symptoms. This questionnaire was chosen to reflect the physical aspect of pain and over time. The FIQ is a 20-item self-reporting questionnaire that assesses symptoms and disability related to FM. The total score ranges from 0 to 100, where a higher score indicates a greater severity of symptoms, ie, lower health status due to FM.^5^

#### 2.2.1. Assessment of pressure pain sensitivity

Pressure pain thresholds (PPTs) were assessed with a hand-held algometer (Somedic Sales AB, Hörby, Sweden), with a probe area of 1 cm^2^ and a pressure increase rate of 50 kPa/s. The subjects were instructed to press a button when the pressure caused the slightest perception of pain. Before the experiment, a few test assessments were conducted in order for the subjects to familiarize with the algometer. Pressure pain thresholds were assessed once at 8 different target sites: bilaterally on m. supraspinatus, the lateral epicondyle of the humerus, the gluteal area, and the inside of the knee (corresponding to the tender points used in the ACR 1990 criteria for FM classification). The average PPT was used for assessment of pain sensitivity.

#### 2.2.2. Assessment of exercise-induced hypoalgesia

**Figure 1.**
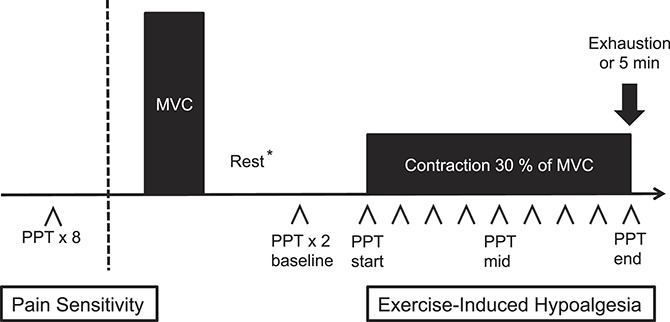
Flowchart of the methodology of the assessment. The pressure pain threshold (PPT) was assessed at 8 body sites. After testing for maximum voluntary contraction (MVC) of m. quadriceps, the subjects performed an isometric contraction of the right m. quadriceps corresponding to 30% of their individual MVC. The PPT at m. deltoideus was assessed before and approximately every 20 seconds during the contraction or for 5 minutes or until exhaustion. *10 to 15 minutes.

The subjects were in a seated position with knee and hip in 90° of flexion. The hips were fixed with a belt and the arms were folded over the chest to minimize body movements. A cuff, connected to a force transducer, was applied around the subject's ankle and fixed to a transverse bar. The maximum voluntary contraction (MVC) in m. quadriceps (MQ) was recorded using Steve Strong (Stig Starke HBI, Göteborg, Sweden), a dynamometer assessing isometric muscle force in the knee extensors.^7^ The subjects were instructed to perform an isometric knee extension by pushing against the cuff with as much force as possible. The MVC was measured 3 times in both legs during a period of 15 seconds with 1 minute of rest between each period. Before the EIH session, the subjects rested for 10 to 15 minutes while doing other noneffort tests (not included in this study). For the experimental part, the subjects were seated in the same position and were instructed to perform a right leg isometric knee extension by pushing against the cuff with the force of 30% of MVC for as long as possible until exhaustion or for a maximum of 5 minutes. The subjects could adjust the force using visual feedback according to the display of Steve Strong. During the experiment, the investigator encouraged the participant to keep the force for as long as possible. Pressure pain thresholds were assessed at the right m. deltoideus (MD) and the contracting MQ first twice ahead of the contraction (the mean used as baseline value), and then continuously during static contraction. One PPT assessment was performed approximately every 20 to 25 seconds depending on how fast the pain threshold was reached, alternating between MD and MQ. Only PPTs assessed at MD were used for the assessment of EIH in this study because they assess the activation of central pain regulatory mechanisms. The values at start, middle (if even number of PPTs, the average of the 2 middle values was calculated), and end of contraction were used for statistics regardless of the total contraction time (Fig. 1).

#### 2.2.3. Genotyping

Saliva samples (Oragene G500) were collected from all subjects and were used for genotyping. All genotyping was performed blind to phenotypic information. The SNPs in this study were chosen based on a priori hypotheses based on existing literature. To be consistent with previous research, the genotypes of the 118A<G SNP *rs1799971* of OPRM1 and the C(-1019)G 5-HT1a promoter polymorphism of *HTR1a* gene were dichotomized into major allele homozygotes and minor allele carriers.^26,42,43^ The 5-HTT gene (*SLC6A4*) comprises the functional polymorphism *5-HTTLPR*, which consists of an L allele and an S allele. The SNP (*rs25531*) containing an _A_-allele and a _G_-allele has been shown to further modulate the efficacy of *5-HTTLPR*. The minor _G_-allele, which almost always coexists with the L-allele, reduces gene expression to S-allele levels. Thus, the functional division of individuals results in a high 5-HTT-expressing group (L_A_/L_A_), an intermediate 5-HTT-expressing group (L_A_/L_G_ or L_A_/S_A_), and a low 5-HTT-expressing group (S_A_/S_A_ or S_A_/L_G_). Studying the 2 polymorphisms jointly, referred to as the triallelic *5-HTTLPR*, is proposed to be more accurate for studying the functionality of the 5-HTT gene.^44^ This has also been confirmed in clinical studies^22^ and was therefore used in this study.

For the polymorphisms *rs1799971* (OPRM1) and *rs6296* (5-HT1a), genotyping was performed using TaqMan SNP genotyping assays and ABI 7900 HT instrument (Applied Biosystems (ABI), Foster City, CA). Polymerase chain reactions (PCRs), with a total volume of 5 μL, were performed in 384-well plates containing 2.5 µL Universal Master Mix (UMM) and 5 ng dried-down genomic DNA per well. The PCR amplification protocol includes 2 holds, 50°C for 2 minutes and denaturation at 95°C for 10 minutes, followed by 45 cycles for *rs6296* and 50 cycles for *rs1799971* at 92°C for 15 seconds and 60°C for 1 minute. For the genotyping of the triallelic *5-HTTLPR*, 2 fragments, 487 bp (short) and 530 bp (long), were amplified by PCRs. Each PCR reaction contained 50 ng DNA, 0.2 mM deoxynucleotide triphosphate (dNTP), 0.4 µM of primer 17P-3F (5′-ggcgttgccgctctgaatgc-3′), 0.4 µM primer 17P-3R (5′-gagggactgagctggacaaccac-3′), 0.05 µL Qiagen HotStar Polymerase, 1 M Q-solution, and finally 1x buffer. Samples were amplified on Biorad Tetrade (BIORAD, Hercules, CA) with an initial denaturation for 10 minutes at 95°C followed by 33 cycles consisting of denaturation for 30 seconds at 95°C, annealing for 30 seconds at 57°C and elongation for 5 minutes at 72°C, and finally followed by another elongation step for 5 minutes at 72°C. Eight microliters of the PCR reactions were separated for 2 hours at 100 V by gel electrophoresis in TBE buffer on a 2.5% agarose gel containing GelRed and visualized using ultraviolet light. To determine the *rs25531*, 10 µL of the PCR product was digested with 0.1 μL MSP1 (New England Biolabs, Ipswich, MA) and 1 μL buffer per sample for 12 hours at 37°C. The MSP1 restriction enzyme breaks the 5′-C/CGG′ sequence that gives a fragment of 342 base pairs, one of 127 and finally one of 62 base pairs which constitutes the L_A_ allele, whereas the 298, 127, and 62 base pairs is the S_A_ allele, the 173, 166, 127, and 62 base pairs for the L_G_ allele, and finally the 166, 130, 127, and 62 for the S_A_ allele. Fragments were run on a 4% agarose gel (3% normal agarose and 1% low melting agarose) containing GelRed initially for 15 minutes at 70 V followed by 2 more hours at 100 V. The gels were then visualized with ultraviolet light.

#### 2.2.4. Statistics

All analyses were performed using SPSS Statistics, version 22.0 (SPSS Inc, Chicago, IL). For all inferences, 2-tailed tests were used and a *P*-value of <0.05 was considered significant. Data were reported as mean ± SD and graphs as mean with error bars of ± 1 SEM. Genotype frequencies were analyzed with the Fisher exact test, and χ^2^ tests were used to assess deviations from Hardy–Weinberg equilibrium. The Shapiro–Wilk test was used to assess the assumption of normality, and when appropriate to use nonparametric tests. To assess differences in pain sensitivity, contraction time, and number of PPT assessments, the Mann–Whitney *U* test was used. Exercise-induced hypoalgesia was analyzed by repeated measures analysis of variance (ANOVA) with the within-subject factor TIME (normalized PPTs; baseline, start, middle, and end) and the between subject factor GROUP (FM or controls) and age as a covariate. The same analysis was also performed with the number of PPT assessments as an additional covariate. Greenhouse–Geisser correction was applied if the assumption of sphericity was violated. All post hoc differences in EIH were analyzed with the Mann–Whitney *U* test, except for group difference over time where the Wilcoxon signed-rank test was used. To assess whether the different genotypes affected symptom parameters, a multivariate ANOVA was performed separately for patients with FM and the HC group. The dependent variables were FIQ, HAD-D, HAD-A, average PPT, and SF-36 BP, the independent variables were the genes OPRM1, 5-HTT, and 5-HT1a, and age was used as a covariate. The overall effects of gene × gene interactions were analyzed by univariate ANOVAs, with a pain modulation score as the dependent variable, group and genotypes as independent variables, and age, HAD-A, and HAD-D as covariates. In addition, the same analysis was performed separately in HC and FM patients with the additional covariates of antidepressants (selective serotonin reuptake inhibitors, serotonin noradrenaline reuptake inhibitors, and tricyclic antidepressants), FM duration, and FIQ in the FM group. This was done to control for more covariates in patients with FM and because the groups differed significantly in the function of EIH and pain modulation scores. Post hoc analyses were performed with univariate ANOVAs and Student *t* test for further analysis of gene interactions.

#### 2.2.5. Normalization of pressure pain threshold and pain modulation score

The interindividual variability in PPTs is pronounced^21^; the range of the values at rest in this study was 29 to 583 kPa. Thus, it was natural to consider the effect on EIH by assessing the relative change in PPTs during contraction, which is fairly constant for each individual.^23^ Therefore, all PPT values were divided by the individual's very first baseline PPT value and are referred to as the normalized PPTs. The normalized PPTs at MD were calculated at baseline, start, middle, and end of each individual's contraction and were used for the analysis of EIH. Furthermore, for genotype effects, the pain modulation score is a quantification of the amount of pain modulation that occurs during the EIH assessment, with zero being no modulation, positive values indicating pain inhibition, and negative values indicating pain facilitation. The pain modulation score was calculated for each individual as the PPT value at the end of contraction minus the mean PPT value at baseline, divided by the mean PPT value at baseline.^28^ The reason for this is to control for individual variation in baseline measures. The score represents the relative difference from baseline PPT value rather than the absolute difference in kPa. However, the pain modulation score was strongly correlated with the absolute change in PPT during contraction (whole group *r* = 0.735, *P* < 0.001; FM *r* = 0.722, *P* < 0.001; and HC *r* = 0.775, *P* < 0.001).

## 3. Results

### 3.1. Participant and genotype characteristics

Patient characteristics grouped by FM patients and HC are presented in Table 1. Compared with HC, patients with FM had significantly lower PPTs and higher ratings of anxiety, depression, and pain. However, the anxiety and depression scores were below 8, ie, the cutoff for the considered presence of anxiety and depressive disease.^6^ Genotype frequencies were similar for all genes for FM patients and HC (Table 2), and allelic frequencies were similar in both groups for all polymorphisms. All polymorphisms were in Hardy–Weinberg equilibrium (OPRM1 *rs1799971 P* = 0.60, 5-HT1a *rs6295 P* = 0.26, 5-HTTLPR *P* = 0.13, 5-HTT *rs25531 P* = 0.20).

**Table 1:**
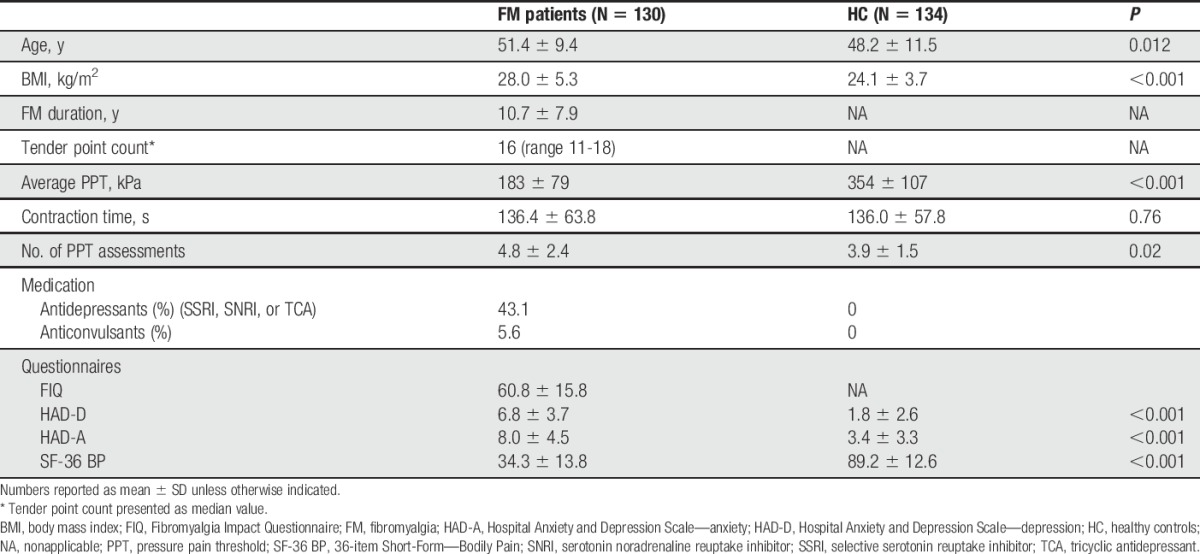
Characteristics of study population, including use of medication and score of standardized questionnaires.

**Table 2:**
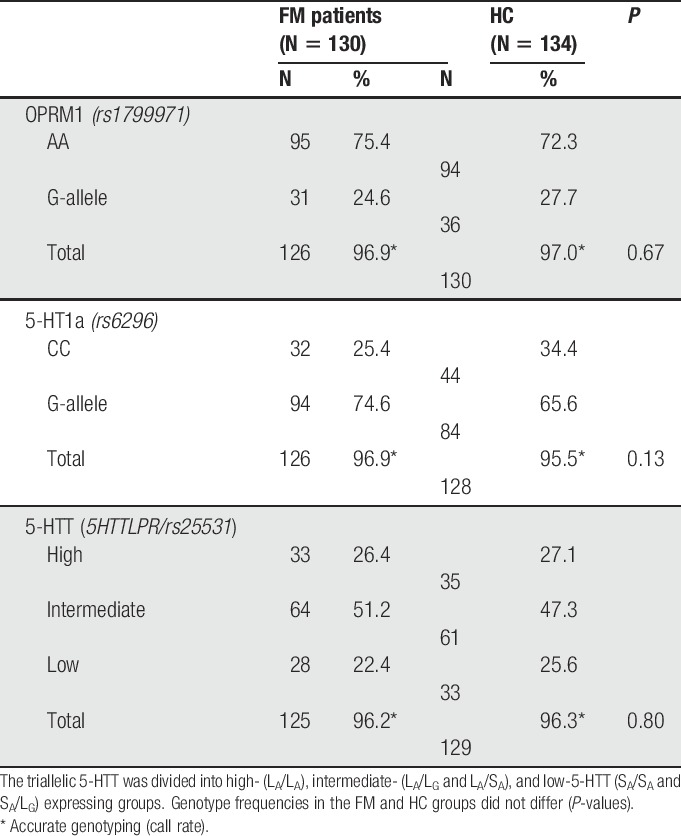
Genotype frequencies of the polymorphisms *rs1799971* (OPRM1), *rs6296* (5-HT1a), and the triallelic 5-HTT for 130 fibromyalgia (FM) patients and 134 healthy controls (HC).

### 3.2. Gene effects on symptoms

Assessments of gene effects on symptoms were performed separately for FM patients and HC because of their different baseline pain level. The ANOVAs revealed no effect of the genetic polymorphisms of OPRM1, 5-HT1a, and 5-HTT on assessed symptom severity (questionnaires FIQ, HAD-A, HAD-D, SF36-BP, and assessment of average PPTs) in either FM patients or HC (FIQ was not analyzed in HC). No significant gene x gene interactions were found regarding symptom severity. Pain sensitivity (average PPT) was increasing with age in both FM (*P* = 0.001) and HC (*P* = 0.049), and SF-36 BP scores were increasing with age (*P* = 0.010), indicating decreasing reports of pain severity with age in patients with FM.

### 3.3. Assessment of exercise-induced hypoalgesia

**Figure 2.**
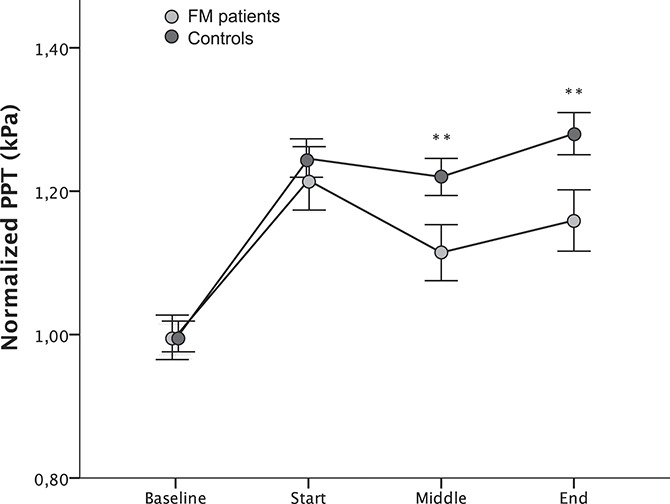
Normalized pressure pain thresholds (PPTs) (mean ± SEM) at baseline, start, middle, and end of a standardized isometric contraction of m. quadriceps corresponding to 30% of maximum voluntary contraction (MVC) assessed at the resting m. deltoideus. There was a significant difference between the groups at middle (*P* = 0.001) and end (*P* = 0.003) of contraction, implying reduced exercise-induced hypoalgesia in patients with fibromyalgia (FM). The curves were adjusted (by adding a coefficient) so that the baseline value always corresponded to 1. Normalized PPT = PPT during contraction/baseline PPT. Statistically significant differences from controls are indicated **P < 0.01.

Regarding the EIH assessment, there was a statistically significant effect of time (*df* = 2.84, F = 5.08, *P* = 0.002), group (*df* = 1, F = 12.18, *P* < 0.001), and a significant time x group interaction (*df* = 2.84, F = 2.85, *P* = 0.040). Because there was a significant difference in the number of PPT assessment, this was assessed as a covariate. The time × group interaction turned out more significant after controlling for the number of PPT assessments (*df* = 2.85, F = 4.04, *P* = 0.008), indicating that the difference is not explained by the difference in the number of PPT assessments. Post hoc analysis revealed that normalized PPTs at MD increased significantly at the end of contraction compared with baseline in patients with FM and controls (both groups *P* < 0.001), indicating functioning pain inhibition in both groups during EIH. Post hoc analysis of group differences during contraction revealed no significant difference at start (*P* = 0.17); however, normalized PPTs were significantly lower in patients with FM at the middle (*P* = 0.001) and the end (*P* = 0.003) of contraction, indicating significantly reduced EIH in patients with FM than controls (Fig. 2). In addition, the mean (SD) pain modulation score, assessing the amount of pain inhibition from baseline to the end of contraction, was 0.09 ± 0.42 in patients with FM and 0.25 ± 0.32 in controls (*P* < 0.001), showing reduced pain inhibitory mechanisms in FM.

### 3.4. Effects of gene-to-gene interactions on pain modulation score

**Figure 3.**
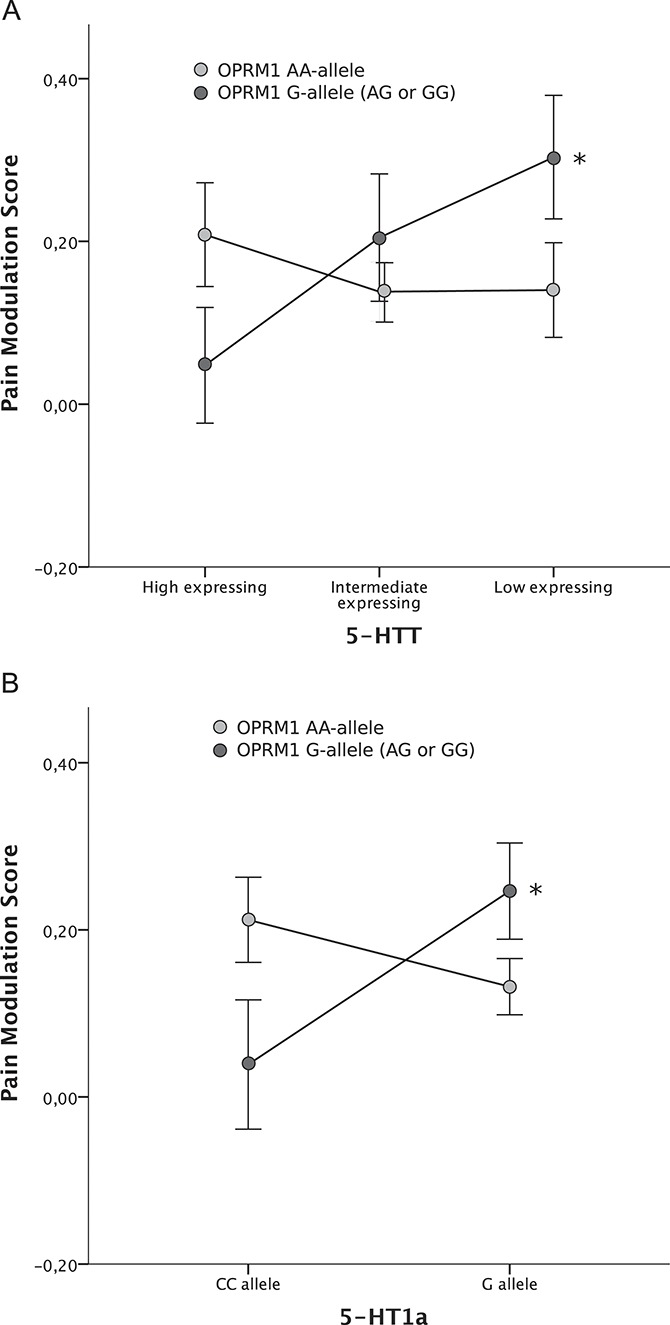
Gene-to-gene interactions between (A) OPRM1 x 5-HTT and (B) OPRM1 x 5-HT1a in fibromyalgia patients and healthy controls grouped together. Assessments were based on the pain modulation score (mean ± SEM), assessing the amount of central pain inhibition during isometric exercise. A significant effect was seen in subjects with OPRM1 G-genotype for both serotonergic genes. Subjects with low-expressing 5-HTT or 5-HT1a G-genotype, respectively, had higher pain modulation scores compared with high-expressing 5-HTT (*P* = 0.023) or 5-HT1a CC-genotype (*P* = 0.037). PPT = pressure pain threshold. Pain modulation score = (PPT end − PPT baseline)/PPT baseline. *P < 0.05.

There were no statistically significant effects on the pain modulation score and no group differences when each polymorphism was tested separately. Neither were there any significant group differences found when analyzing the effects of gene-to-gene interactions on the pain modulation score. There was a statistically significant interaction between OPRM1 and 5-HTT (*df* = 2, F = 3.19, *P* = 0.043), and a significant effect for the covariate anxiety (HAD-A) (*df* = 1, F = 4.45, *P* = 0.036). Post hoc analysis showed that individuals with OPRM1 G-genotype in combination with genetically inferred 5-HTT low expression had higher pain modulation scores compared with 5-HTT high expression (pain modulation scores: 5-HTT low = 0.30 and 5-HTT high = 0.048; *P* = 0.023), indicating better central pain inhibition (Fig. 3A). In analogy with 5-HTT, a similar interaction was seen between OPRM1 and 5-HT1a (*df* = 1, F = 4.55, *P* = 0.034), but no significant effects of covariates were found. Post hoc analyses revealed that in individuals with OPRM1 G-genotype, also having the 5-HT1a G-genotype, yielded significantly higher pain modulation scores compared with 5-HT1a CC-genotypes (pain modulation scores: CC = 0.039 and G = 0.25; *P* = 0.037), indicating better central pain inhibition with the genetic setup of OPRM1 G-genotype and 5-HT1a G-genotype (Fig. 3B). Strengthening the patterns in Figure 3, there were some trends that were nearly significant. In the 5-HT1a CC-genotype group, the pain modulation score was higher in OPRM1 AA-genotypes compared with OPRM1 G-genotypes (*P* = 0.070). In the 5-HT1a G-genotype group, the pain modulation score was higher for OPRM1 G-genotypes compared with OPRM1 AA-genotypes (*P* = 0.097). No significant interaction between 5-HTT and 5-HT1a was found, and thus, no post hoc analyses were performed.

### 3.5. Effects of gene-to-gene interactions on pain modulation score in fibromyalgia and healthy controls separately

To control for additional relevant covariates in the FM group, such as antidepressant medication (selective serotonin reuptake inhibitors, serotonin noradrenaline reuptake inhibitors, or tricyclic antidepressants), FM duration, and FM severity (FIQ), the analyses were performed separately for FM and HC. No statistically significant main effects on the pain modulation score were found for the individual genetic polymorphisms when tested separately in patients with FM. The analysis showed a statistically significant gene-to-gene interaction between OPRM1 and 5-HTT in patients with FM (*df* = 2, F = 3.38, *P* = 0.038). This analysis demonstrated an effect of the covariate HAD-A in patients with FM (*df* = 1, F = 4.00, *P* = 0.048), but there was no effect of FM duration, FM severity, or the use of antidepressant medication.

**Figure 4.**
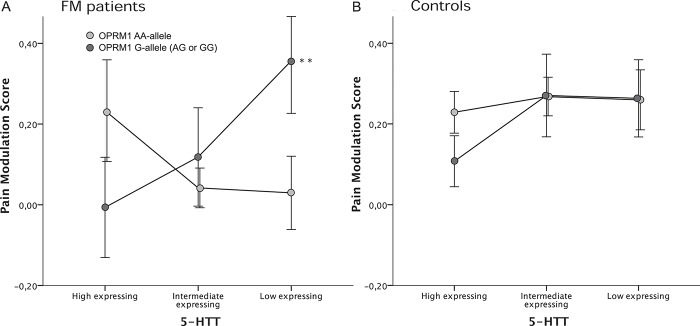
Gene-to-gene interactions between OPRM1a and 5-HTT in (A) patients with fibromyalgia (FM) and (B) healthy controls when assessing exercise-induced hypoalgesia. A significant interaction was found in patients with FM (*P* < 0.05)—subjects with OPRM1 G-genotype had a significantly higher pain modulation score if they also were genetically inferred 5-HTT low expressing compared with 5-HTT high expressing (*P* < 0.01). In accordance, the analysis exhibited a trend in low-expressing 5-HTT carriers where OPRM1 G-genotype conferred a higher pain modulation score than AA-genotype (*P* = 0.10). No significant interactions were found in the control group. PPT = pressure pain threshold. Pain modulation score = (PPT end − PPT baseline)/PPT baseline.

Further post hoc analysis revealed that the difference was specific for OPRM1 G-genotypes, with significantly higher pain modulation scores in FM patients with genetically inferred 5-HTT low expression compared with high expression (pain modulation scores: 5-HTT low = 0.35, 5-HTT high = −0.006; *P* = 0.008). Furthermore, a trend was found in carriers of the 5-HTT low genotype where the OPRM1 G-genotype had a higher pain modulation score than AA-genotype (*df* = 1, F = 2.93, *P* = 0.10) (Fig. 4A). No significant interactions were found in the HC group (Fig. 4B).

**Figure 5.**
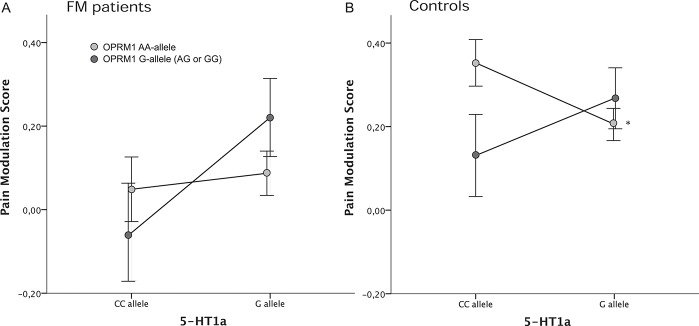
Gene-to-gene interactions between OPRM1a and 5-HT1a in (A) patients with fibromyalgia (FM) and (B) healthy controls when assessing exercise-induced hypoalgesia. There was a significant interaction of OPRM1 and 5-HT1a in the healthy control group (*P* < 0.05). OPRM1 AA-genotypes had a significantly higher pain modulation score if they also were 5-HT1a CC-carriers compared with G-carriers (*P* < 0.05). A trend supporting the results was exhibited for 5-HT1a CC-genotypes, who had a higher pain modulation score if they were OPRM1 AA-carriers compared with G-carriers (*P* = 0.059). PPT = pressure pain threshold. Pain modulation score = (PPT end − PPT baseline)/PPT baseline.

The OPRM1 x 5-HT1a analyses revealed a statistically significant gene-to-gene interaction in the HC group (*df* = 1, F = 4.33, *P* = 0.040). Post hoc analysis revealed that the significance was specific for the OPRM1 AA-genotype, with a higher pain modulation score in individuals with the 5-HT1a CC-genotype compared with 5-HT1a G (pain modulation scores: 5-HT1a CC = 0.35, 5-HT1a G = 0.20; *P* = 0.045). There was a trend that HC with 5HT1a CC-genotype had a higher pain modulation score if they were carriers of the OPRM1 AA-genotype compared with G-genotype (pain modulation scores: OPRM1 AA = 0.35, OPRM1 G = 0.13; *P* = 0.059) (Fig. 5B). No significant main effects or gene-to-gene interactions were seen in the FM group (Fig. 5A), nor were any significant effects of the covariates found.

## 4. Discussion

This study examined the role of specific functional genetic polymorphisms, acting on opioid and serotonergic signaling, on EIH in healthy subjects and patients with FM. None of the polymorphisms of OPRM1, 5-HTT, or 5-HT1a had an effect on EIH on their own. The major findings in both groups were the significant interactions between functional polymorphisms in the genes OPRM1 x 5-HTT and OPRM1 x 5-HT1a, suggesting joint effects of opioid and serotonergic mechanisms regulating central pain inhibitory signaling. Individuals with OPRM1 G-genotype in combination with either low expression of 5-HTT or the 5-HT1a G-genotype seemed to be helped by their genetic setup so that they had a better functioning endogenous pain inhibition compared with other serotonergic genotypes (5-HTT high or 5-HT1a CC). Although it has been shown that opioids, via disinhibition, engage inhibitory 5-HT projections to produce antinociception,^4^ animal research has found an antagonistic effect between mu-opioid and 5-HT1a receptor activation.^8,9^ More precisely, mu-opioid agonists have short-term analgesic effects followed by opioid hyperalgesia, whereas 5-HT1a receptor agonists have short-term hyperalgesic effects followed by analgesia. In addition, 5-HT1a receptor agonists have been reported to reverse opioid hyperalgesia and tolerance.^8,9^ In line with this, based on the proposed mechanisms for the studied polymorphisms,^33,37,44^ our results imply that a person's ability to activate central pain inhibition is better if their opioid system is genetically inferred to increase the endogenous opioid tone (OPRM1 G-allele) while at the same time reducing serotonergic signaling (low-5-HTT expression or 5-HT1a G-allele). Thus, the joint genetic effect of OPRM1 x 5-HTT and OPRM1 x 5-HT1a on the pain inhibitory pathways tends to be working antagonistically, as our hypothesis stated. The fact that both serotonergic genotypes that mechanistically are proposed to reduce 5-HT transmission (5-HTT low and 5-HT1a G) independently interacted with OPRM1 in a similar direction (OPRM1 G) further validates the hypothesis of antagonistic opioid x 5-HT interactions on pain modulatory mechanisms.

### 4.1. No effects of single functional polymorphisms on exercise-induced hypoalgesia

That both opioids and serotonin are involved in the endogenous pain modulatory system interactions is generally accepted.^35^ However, few studies have examined the association between polymorphisms in opioid and serotonin-related genes and endogenous pain modulation. We found no significant associations between the studied polymorphisms of OPRM1, 5-HTT or 5-HT1a, and EIH when they were studied separately. Previous studies showed that healthy individuals with 5-HTT low expression had decreased conditioned pain modulation^28^ and decreased sensory modulation.^40^ However, conditioned pain modulation and EIH are 2 different paradigms to study pain modulatory processes and they do not seem to be correlated.^14,41^ Thus, the genetic associations related to alterations in EIH seem to be only noticeable when considering the interaction of more than 1 polymorphism, stressing the importance of studying joint gene effects on pain modulation.

### 4.2. Effects of gene-to-gene interactions on exercise-induced hypoalgesia

In this study, we found significant interactions between the OPRM1 G-allele and both serotonergic gene variants proposed to reduce 5-HT signaling. Whereas the results were clear in this direction the contrary, meaning that genetically inferred reduced opioid tone combined with increased serotonergic signaling (ie, OPRM1 AA and 5-HTT high/5-HT1a CC) jointly yields better functioning EIH, was less consistent. The only statistically significant finding in this direction was that healthy individuals with OPRM1 AA had higher pain modulation scores if they had the 5-HT1a CC compared with the 5-HT1a G-genotype. It is worth noticing that all analyses that reached significance did so in the direction in line with our hypothesis regarding HC; strong opioids together with weak 5-HT or weak opioids together with strong 5-HT seemed to provide better pain inhibition during exercise.

The 5-HT1a G variant exerts dual effects on 5-HT1a receptor expression depending on where the receptor is situated. It yields upregulation of inhibitory autoreceptors in the raphe nuclei but downregulation of postsynaptic 5-HT1a receptors in the projection areas.^11^ Thus, the total synergistic effect of 5-HT1a G has decreased 5-HT transmission both in raphe nuclei and its peripheral projection areas.^37^ Interestingly, the 5-HTT low-expressing genotype has been proposed to downregulate 5-HT1a receptors.^12,30^ Thus, in the raphe nuclei 5-HT-synthesizing neurons, the 5-HT1a G-genotype upregulates 5-HT1a receptors, reducing 5-HT transmission, whereas the 5-HTT low genotype conversely downregulates 5-HT1a receptors, increasing 5-HT transmission. On the contrary, in the postsynaptic neurons, both the 5-HT1a G and 5-HTT low would be expected to downregulate 5-HT1a receptors, reducing 5-HT1a-mediated 5-HT effects. Thus, 5-HTT low and 5-HT1a G should have the same physiological effect in postsynaptic nonserotonergic neurons but opposing effects in the serotonergic neurons of raphe nuclei. Therefore, our results showing similar OPRM1 x 5-HTT low and OPRM1 G x 5-HT1a G interactions regarding EIH would indicate that the effect is mediated by postsynaptic nonserotonergic neurons in the projection areas rather than by the 5-HT-synthesizing neurons in the raphe. Interestingly, greater availability of 5-HT1a receptors in brain areas associated with pain processing, including dorsal raphe nuclei and its projection areas, has been related to greater ability to suppress pain.^32^ If greater availability indicates more 5-HT1a-mediated 5-HT inhibition, and thus reduced 5-HT transmission, then this corresponds to the proposed mechanism of the 5-HT1a G variant and is accordingly in line with our results.

### 4.3. The effects of gene-to-gene interactions on exercise-induced hypoalgesia did not differ between fibromyalgia patients and healthy controls

In accordance with previous research, patients with FM had reduced function of EIH compared with HC.^20,24,38^ Despite this, we found no overall significant differences between the groups regarding the gene-to-gene interactions on EIH. Patients with FM showed interactions in the same direction as HC—individuals with genetically inferred stronger endogenous opioid signaling (OPRM1 G) and weaker serotonergic signaling (5-HTT low/5-HT1 G) had better pain modulation scores compared with serotonin strong signaling (5-HTT high/5-HT1a CC). In addition, several trends support that FM and HC exhibit opioid x 5-HTT and opioid x 5-HT1a interactions in the same direction. Thus, we found no support for our hypothesis of different opioid x 5-HT interaction patterns as the basis for the reduced function of EIH in patients with FM. The results are in accordance with the absence of gene-to-gene interactions to explain the difference in FM symptoms between patients and HC, ie, average PPT and questionnaires FIQ, HAD-A, and HAD-D.

### 4.4. Limitations

First, there was a minimal, yet statistically significant difference in age, which likely does not affect the results. Moreover, although all subjects were whites, a few in each group had ethnicities outside Europe. These subjects did not differ in allelic frequencies from the whole group; thus, it is not likely that population stratification has a significant effect on the results. Also, the functions of the mu-opioid receptor, the serotonin transporter, and the 5-HT1a receptor were inferred from genotypes and not assessed directly. However, the method is well established in the literature and allows us to study the effects on behavior under normal conditions, ie, without pharmacological manipulation. Furthermore, based on previous studies, we examined the effects of 4 polymorphisms within 3 genes. This does not exclude that other genetic polymorphisms are in linkage and influence gene expression and transcription, nor are epigenetic changes taken into account. However, the polymorphisms studied were thoroughly chosen based on previous research demonstrating their involvement in pain regulation.

## 5. Conclusions

Our results suggest that, by genetic association, the mu-opioid receptor interacts with 2 major serotonergic structures involved in 5-HT reuptake and release, to modulate EIH. Furthermore, we found that the interaction worked in an antagonistic manner, ie, genetically inferred increased opioid signaling combined with decreased serotonergic signaling produced better pain inhibition during exercise. Lastly, in contrast to our a priori hypothesis, the opioid x 5-HT interactions on pain modulation existed regardless of baseline function of endogenous pain modulatory mechanisms, with similar effects in patients with FM and HC. Thus, our results do not support an altered interaction between opioid and 5-HT mechanisms as the basis for dysfunction of EIH in FM. Instead, we were able to reproduce similar findings of interactions between opioid x serotonergic signaling in 2 different human cohorts.

As no effect of a singular genetic polymorphism was found, the present results indicate the importance of assessing joint gene effects when studying behavioral traits with complex modulatory mechanisms, such as pain modulation. To our knowledge, no previous reports have been made on genetic interactions between the opioid and the serotonergic systems on pain mechanisms in humans. Many current pain medications target either the opioid system or the serotonergic system; therefore, increased understanding of the interactions between the systems could help develop new combined treatment options of pain syndromes.

## Conflict of interest statement

The authors have no conflicts of interest to declare.
